# Numerical study of the electroporation pulse shape effect on molecular uptake of biological cells

**DOI:** 10.2478/v10019-010-0002-3

**Published:** 2010-03-18

**Authors:** Damijan Miklavcic, Leila Towhidi

**Affiliations:** Faculty of Electrical Engineering, University of Ljubljana, Ljubljana, Slovenia

**Keywords:** electrochemotherapy, optimization, membrane permeability, membrane conductivity

## Abstract

**Background:**

In order to reduce the side-effects of chemotherapy, combined chemotherapy-electroporation (electrochemotherapy) has been suggested. Electroporation, application of appropriate electric pulses to biological cells, can significantly enhance molecular uptake of cells due to formation of transient pores in the cell membrane. It was experimentally demonstrated that the efficiency of electroporation is under the control of electric pulse parameters. However, the theoretical basis for these experimental results is not fully explained. In order to predict the outcome of experiments and optimize the efficiency of electroporation before each treatment, we developed a model to investigate the effect of pulse shape on efficiency of electroporation.

**Results:**

Our model is based on a developed chemical-kinetics scheme and trapezium barrier model, while self-consistency was taken into account. This model is further supplemented with a molecular transport model to acquire the molecular uptake of cells. The investigated pulse shapes in this study were unipolar rectangular pulses with different rise and fall times, triangular, sinusoidal and bipolar rectangular pulses and also sinusoidal modulated unipolar pulses with different percentages of modulation. The obtained results from our modelling and simulations are in good agreement with previously published experimental results.

**Conclusions:**

We therefore conclude that this model can be used to predict the effects of arbitrarily shaped electroporation pulses on cell membrane conductivity and molecular transport across the cell membrane.

## Introduction

Cancer is a leading cause of death around the world and for this reason it has drawn the attention of many researchers. Chemotherapy has been used for many years and is one of the most common treatments for cancer. Cytotoxic chemotherapeutic drugs are usually hydrophilic with very low transport through the cell membrane and thus high doses of these drugs are needed for treatment. Therefore, while chemotherapy can be quite effective in treating certain cancers by interfering with the cancer cell’s ability to grow or reproduce, chemotherapeutic drugs reach all parts of the body, not just the cancer cells. Because of this, there may be many side-effects such as nausea, blood cell deficiency, fatigue and loss of hair during treatment.^1^

Two decades ago, electrochemotherapy was suggested for its use in clinical treatment of localized tumors.^2^ Electrochemotherapy consists of electroporation and chemotherapy. Electroporation is a technique in which permeability of the plasma membrane increases transiently and reversibly with appropriate pulse parameters^3^–^6^ and is nowadays widely used not only in electrochemotherapy,^7^–^9^ but also in biotechnology^10^,^11^ and in medical applications such as gene electrotransfer^12^,^13^ and transdermal drug delivery.^14^ Electroporation in combination with chemotherapy can increase drug delivery into the cells and consequently drug doses and thus side-effects of chemotherapy can be reduced.^10^, ^11^,^15^,^16^

It was experimentally demonstrated in a number of studies that the efficiency of electroporation is under the control of electric pulse parameters such as pulse amplitude, duration, and shape.^17^–^22^ Optimization of electric field parameters for successful electroporation requires time-consuming and costly experiments for different experimental criteria unless an appropriate model for this phenomenon can be suggested. Although the known models were proposed in previous studies^23^–^28^, they are still unable to explain the effect of some parameters such as pulse shape, pulse repetition frequency and number of pulses on the molecular uptake enhancement of the cells under exposure.

The suggested mechanism for electroporation consists of structural changes resulting in formation of transient aqueous pores in the cell membrane. In order to reveal the exact mechanisms and dynamics of pore formation and closure and more importantly resealing of the membrane, theoretical models have drawn a great deal of attention.

In our present study, we investigated the effect of pulse shape on the efficiency of electroporation and, therefore, electrochemotherapy using modelling and simulation. Our model was based on a chemical-kinetics scheme with two types of pores^29^ which have been recently confirmed.^30^ We used developed equations with field-dependent rate coefficients in order to obtain the pore distribution on the membrane. Besides, the conductivity of pores was defined based on a trapezium barrier model for the image forces.^31^ A self-consistent set of equations was used to consider all simultaneous changes. This model was supplemented with a molecular transport model for a single cell to acquire the molecular uptake of cells. The investigated pulse shapes in this study were unipolar rectangular pulses with different rise and fall times, triangular, sinusoidal and bipolar rectangular pulses and also sinusoidal modulated unipolar pulses with different percentages of modulation – all previously used in experimental studies.

## Material and methods

### Model description

#### Modified chemical-kinetics model for electroporation

When a cell is exposed to an external electric field, the induced transmembrane voltage (ITV) starts to increase based on the Laplace equation which leads to structural changes of the cell membrane. Based on a previously suggested^29^, and recently confirmed^30^ kinetic model, in the first step the intact closed lipids (C) transform to tilted lipid headgroups (C1). In the second step, the prepores (P1) are formed and finally, in the last step the final pores (P2) are formed. The sequential reaction can be described by:

[1] $$\text{C}\overset{{\text{k}}_{1}}{\underset{{\text{k}}_{-1}}{⇄}}{\text{C}}_{1}\overset{{\text{k}}_{2}}{\underset{{\text{k}}_{-2}}{⇄}}\text{P1}\overset{{\text{k}}_{3}}{\underset{{\text{k}}_{-3}}{⇄}}\text{P}2$$

The permeability of the P1 state is negligibly small and P2 is predominantly responsible for molecular uptake. Pore formation and closure are denoted by k_i_ and k_−i_ (i=1,2,3) rate coefficients, respectively. For simplicity, the rate coefficients k_1_, k_2_ and k_3_ are considered equal (k_1_=k_2_=k_3_=k_p_).^29^ The governed rate laws of constituting steps for the scheme [1] are:

[2] $$\begin{array}{l} \frac{d[C(\vec{r},t)]}{dt}=-k_{p}[C(\vec{r},t)]+k_{-1}[C1(\vec{r},t)]\\ \frac{d[C1(\vec{r},t)]}{dt}=-k_{p}([C1(\vec{r},t)]-[C(\vec{r},t)])-k_{-1}[C1(\vec{r},t)]+k_{-2}[P1(\vec{r},t)]\\ \frac{d[P1(\vec{r},t)]}{dt}=-k_{p}([P1(\vec{r},t)]-[C1(\vec{r},t)])-k_{-2}[P1(\vec{r},t)]+k_{-3}[P2(\vec{r},t)]\\ \frac{d[P2(\vec{r},t)]}{dt}=k_{p}[P1(\vec{r},t)]-k_{-3}[P2(\vec{r},t)] \end{array}$$

where t and *r⃗* denote time and position, respectively. [C], [C1], [P1] and [P2] show normalized distribution of each membrane lipid state relative to the initial value of the closed state [*C*(*r⃗*,0)].^29^

Regarding the Van’t Hoff relationship in electro-thermodynamics, the rate coefficient of pore formation can be obtained from:^29^,^31^

[3] $$k_{p}=k_{p0}\text{exp}\left(\frac{\Delta V_{p}\varepsilon_{0}(\varepsilon_{W}-\varepsilon_{L})}{2k_{B}Td_{m}{}^{2}}ITV^{2}\right)$$

where ITV is the potential difference between the outer and inner layer of the membrane, Δ*V_p_* is the mean volume change due to pore formation, *ɛ*_0_ is the permittivity of the vacuum and *ɛ_W_* and *ɛ_L_* are dielectric constants of water and lipids, respectively. k_B_ is the Boltzmann constant, d_m_ is the thickness of the membrane and T is temperature. While the pore formation rate coefficient *k_p_* is electric field-dependent, the closure rate coefficients (k_−1_, k_−2_ and k_−3_) are constant and independent of electric field strength.^29^

Whenever electroporation occurs, an increase in conductivity during the pulse is observed^32^ which can be explained by the formation of pores in the cell membrane. Based on the trapezium barrier model for the image forces, the intrinsic pore conductivities *σ_p,i_* (i=1 and 2 represents P1 and P2 pores, respectively) are expressed as follows:^31^,^33^

[4] $$\sigma_{p,i}=\sigma_{p,i}^{0}\text{exp}\left(\alpha_{p,i}n\left|ITV\right|\frac{F}{RT}\right)$$

where

[5] $$\sigma_{p,i}^{0}=\frac{\sigma_{\mathrm{ex}}+\sigma_{\mathrm{in}}}{2}\text{exp}\left(\frac{-\varphi_{\mathrm{im},i}^{0}F}{RT}\right)\;\;\;\;\;\;\text{and}\;\;\;\;\alpha_{p,i}=1-\frac{RT}{F\varphi_{\mathrm{im},i}^{0}}$$

In the above equations, *σ_ex_* and *σ_in_* are the extracellular and intracellular conductivities respectively, n is the geometrical parameter of the trapezium model for energy barrier, F is the Faraday constant and $\varphi_{\mathrm{im},i}^{0}$ is the intrinsic pore barrier potential.

Therefore, conductivity of the membrane (*σ_m_*) can be obtained by:

[6] $$\sigma_{m}(\vec{r},t)=\sigma_{m0}+\left[P1(\vec{r},t)\right]\times \sigma_{p,1}+\left[P2(\vec{r},t)\right]\times \sigma_{p,2}$$

where *σ*_0_ is the physiological/baseline conductivity of the membrane. Thus conductivity at each point on the membrane changes with time during and after the pulse, depending on pore distribution variations which affect ITV and in turn the distribution of pores.

#### Transmembrane molecular transport model

Based on previous studies^34^–^36^, we defined two distinct phases for the electroporated membrane and two related transport mechanisms: the first one is the porated phase [P2] with relatively fast relaxation due to pore closure according to Eq. [1]. The second phase is the memory phase [M] due to enhanced membrane perturbation and ruffling with quite slow relaxation^34^–^36^ which returns to its baseline value with a dual exponential decay function:^29^

[7] $$[M]={\left[P2\right]}_{e}\left(B\text{exp}(-k_{f}t)+(1-B)\text{exp}(-k_{s}t)\right)$$

where [*P*2]*_e_* is the normalized distribution of [P2] pores at the end of the pulse, k_f_ and k_s_ are decay rate coefficients for this second phase and B is a constant.

The considered transport mechanisms for these two phases were interactive diffusion through the pores and endocytotic-like transport through the permeabilized area of the membrane.^34^–^37^ Thus, the permeability of the membrane can be written as the sum of two distinct contributions:

[8] $$P_{m}(\vec{r},t)=\left(\left[P2(\vec{r},t)\right]D_{p}/d_{m}\right)+\left(\left[M(\vec{r},t)\right]D_{r}/d_{m}\right)$$

where D_p_ and D_r_ are the attributed diffusion coefficients for interactive transport and endocytotic-like transport, respectively.

While the membrane is being permeabilized due to the electric field, the molecules pass through the membrane due to a concentration gradient. A quantitative description of diffusion is contained in Fick’s first law. The total flux can be approximated by *j* = *P_m_* (*c^out^* – *c^in^*), where *c^out^* and *c^in^* are the outer and inner concentrations adjacent to the membrane. The total number of molecules transported through the membrane (N) was computed with integration of transported molecules through the cell membrane over time and the cell surface:

[9] $$N=N_{A}\int_{t=0}^{\tau }\int_{S}jdSdt$$

where S is the surface of the cell membrane, *τ* is the time at which the quantity of transported molecules is to be determined and *N_A_* is Avogadro’s number.

### Construction of the model

The simulations in this study were performed using the COMSOL 3.3 package (COMSOL Inc., Burlington, MA) based on the finite element method. To construct the geometrical model, a spherical cell with radius of 5.6 μm was located between two virtual electrodes. Since incorporating an extremely thin membrane is problematic in meshing and solving the problem, we assigned the boundary condition to the membrane.^38^ We neglected the resting transmembrane voltage. The initial intracellular and extracellular concentrations of probe were set to 0 and 10 mM, respectively. The diffusion coefficients for interactive diffusion and for an induced endocytotic-like process are considered as D_0_/5 and D_0_/10000. These two values, however, depend considerably on the type and size of the transported molecules. The necessary parameters used in our simulations are given in Table 1. Our simulation was designed to solve the Laplace equation considering all related equations in this model (Eq. [2], [3], [6]) taking into account self-consistency of parameters to find the distribution of pores on the cell membrane, spatially and temporally, and all related parameters such as ITV, cell membrane conductivity and permeability. Afterwards, the up-take of the cells for each different pulse shape was obtained. All simulations were performed on a PC (2.8 GHz Pentium IV processor, 3 GB RAM) and each simulation lasted 3–25 minutes depending on the considered pulse shape and number of pulses in each train of pulse.

**FIGURE 1:**
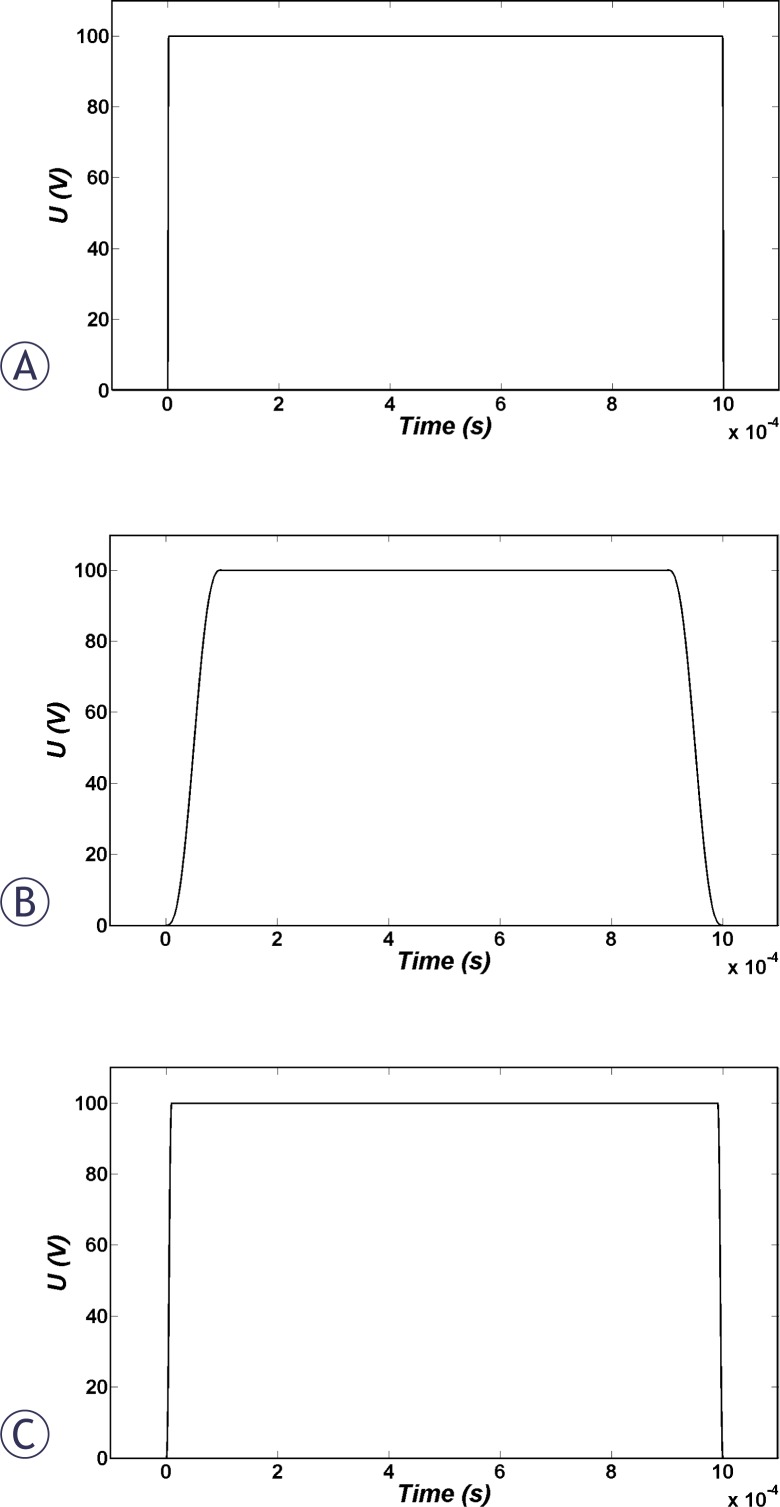
The investigated rectangular pulse shapes in this study with rise and fall times of (A) 2, (B) 10 and (C) 100 μs, respectively.

**FIGURE 2:**
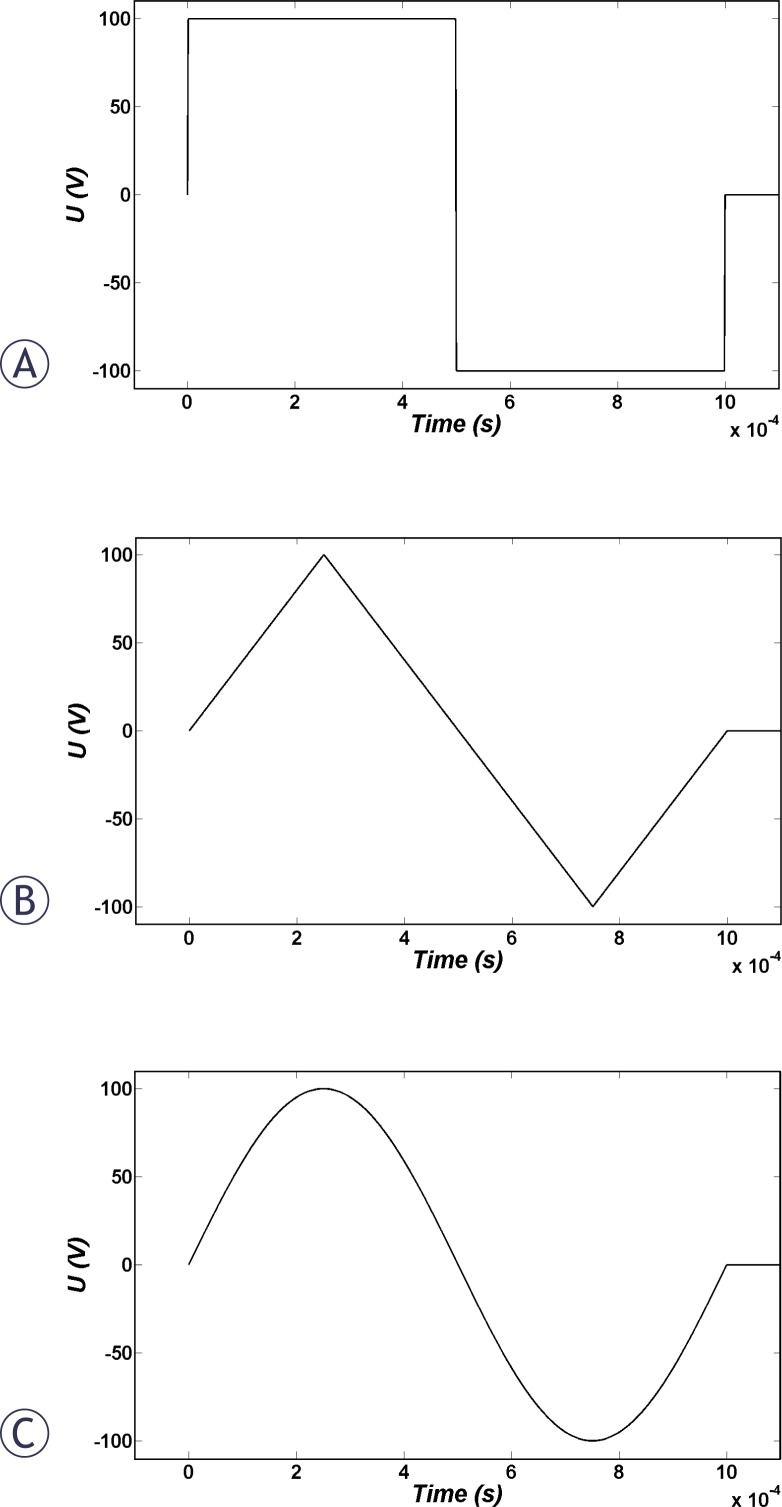
The (A) bipolar rectangular, (B) triangular and (C) sinusoidal pulses considered in this study.

**FIGURE 3:**
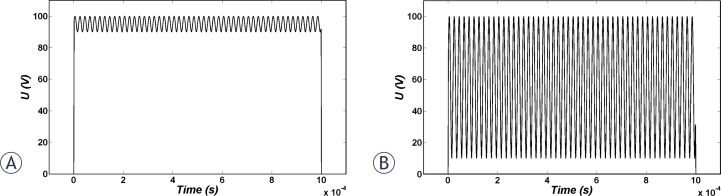
The sine-modulated 50 kHz unipolar pulses with (A) 10% and (B) 90% modulation investigated in this paper.

The investigated pulse shapes in this study were unipolar rectangular pulses with different rise and fall times of 2, 10 and 100 μs (Figure 1); triangular, sinusoidal and bipolar rectangular pulses (Figure 2); and also sinusoidal modulated unipolar pulses with different percentages of modulation of 10% and 90% with 50 kHz frequency (Figure 3).

## Results

**FIGURE 4:**
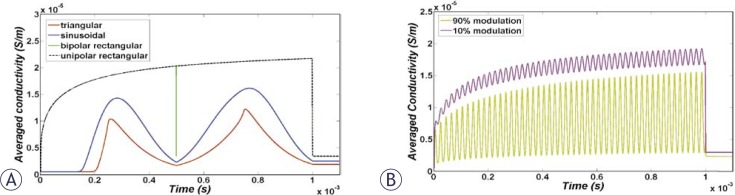
Temporal evolution of the overall membrane conductivity during the pulse for (A) unipolar and bipolar rectangular, triangular and sinusoidal pulses and (B) 10% and 90% sine-modulated unipolar pulses.

Immediately after the smoothed step pulse is switched on, ITV starts to increase based on the Laplace equation and causes membrane structural changes initiation, which in turn results in the membrane conductivity increase according to Eq. [6]. The temporal behaviour of average conductivities over the cell membrane due to application of the considered pulse shapes (Figures 1, 2 and 3) are shown in Figure 4. All pulses were considered to have a peak of 1 kV/cm and total duration of 1 ms.

It can be observed in Figure 4 that the overall conductivity changes for unipolar and bipolar pulses have negligible differences. The reason for this fact is a very quick switch between positive and negative voltage, as well as ignoring the resting voltage in this model. Besides, a comparison between conductivity increases due to rectangular, triangular and sinusoidal pulses was performed. Figure 4A shows that the largest and smallest changes were due to rectangular and triangular pulse shapes, respectively. Figure 4B shows that a conductivity change due to 10% modulation is higher than the 90% one but both are still lower than the rectangular pulse.

**FIGURE 5:**
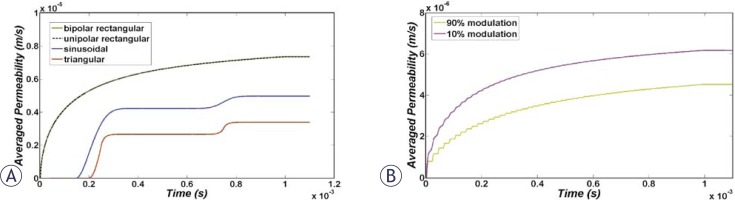
Temporal evolution of the overall membrane permeability during the pulse for (A) unipolar and bipolar rectangular, triangular and sinusoidal pulses and (B) 10% and 90% sine-modulated unipolar pulses.

The temporal behaviour of averaged cell membrane permeability for pulses in Figures 1 to 3 is illustrated in Figure 5. Permeability changes occur slowly. Therefore, for bipolar pulses and modulated pulses in which the fall and rise is very fast, there is not enough time for resealing of permeability which causes different behaviour for membrane permeability related to membrane conductivity. Based on Figure 5, we expect the order of efficiency of pulses of the same peak as follows: unipolar and bipolar rectangular, 10% modulated, sine, 90% modulated and finally bipolar triangular pulses. Note that unlike membrane conductivity in Figure 4, membrane permeability does not recover as fast after the pulse ceases.

To be able to check the validity of our simulation results, the uptake enhancement of the cell was calculated for the same pulse parameters of previously obtained experimental results.^19^ The chosen parameters were 8 pulses of 100 μs duration and 1 Hz pulse repetition frequency with different pulse strengths for each pulse shape.

**FIGURE 6:**
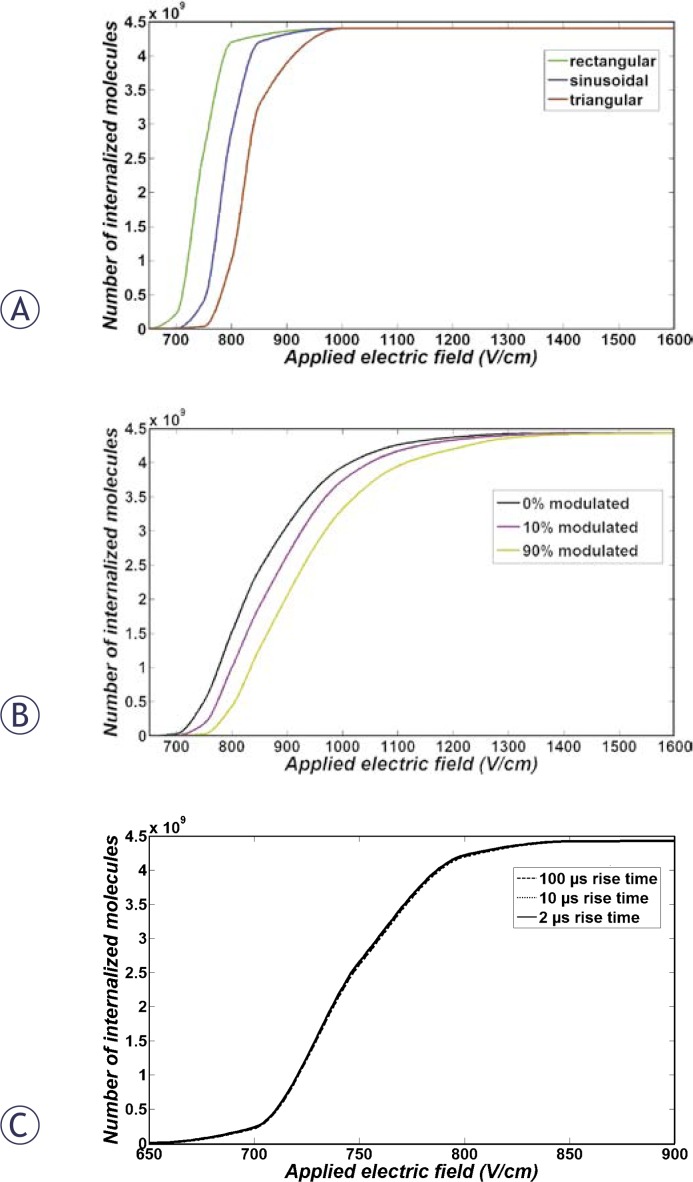
Dye uptake 16 minutes after pulse cessation versus electric field amplitude for (A) 8 pulses of 1 ms and 1 Hz unipolar and bipolar rectangular, triangular and sinusoidal pulses, (B) one 1 ms pulse of 0%, 10% and 90% sine-modulated and (C) 8 pulses of 1 ms rectangular pulses with rise and fall times of 2, 10 and 100 μs.

Figure 6A shows the results of simulation for 8 pulses of bipolar rectangular, sine and triangular pulses. It shows that the rectangular pulses are more efficient than sinusoidal pulses which in turn are more efficient than triangular pulses. These results are in good agreement with experimentally obtained results.^19^

As can be seen in Figure 2A for the bipolar pulses, the pulse switch from positive to negative takes place very fast. During the switch time, the pore creation rate and, therefore, membrane conductivity decrease. But due to very short time of switching related to pulse duration, these changes are negligible in comparison to the conductivity change related to the unipolar pulse (Figure 4A). Consequently, the uptake due to unipolar pulses is larger than bipolar pulses but this difference is negligible and not observable (data not shown). While our simulation shows no significant difference between these two pulse types, in experimental results bipolar pulses are significantly more efficient than unipolar pulses. The reason for this inconsistency is most probably due to neglecting resting voltage in the simulations.

In addition, Figure 6B demonstrates the comparison between unipolar pulses of 0, 10 and 90% modulation. The results are also in good qualitative agreement with previously obtained experimental results.^19^ The uptake enhancement results for 8 pulses of unipolar trapezoidal pulses of 1 ms duration with 2, 10 and 100 μs rise and fall times are shown in Figure 6C. It can be seen from the figure that there is no significant difference between these pulses which is again in good agreement with previously published experimental results.^19^

## Conclusions

The described model enables determination and prediction of all electrical and diffusion parameters for different pulse shapes. Thus, knowing electrical and diffusion properties of the cells and the specific dye, optimization of the electroporation protocol can be performed before the treatment. Our results show that rectangular pulses are more effective than the sinusoidal and triangular pulses. Besides, our results indicate that the higher the percentage of unipolar pulses modulation with sine shape pulses of 50 kHz, the lower the uptake enhancement of the cells. Moreover, the rise and fall times of unipolar rectangular pulses do not significantly affect the uptake of molecules by the cells. Our simulation results are consistent with experimental observations.

## Figures and Tables

**TABLE 1:** Values of parameters used in simulations

**Parameter**	**Symbol**	**Value**	**Ref.**
Membrane thickness	d_m_	5e–9 m	29
Extracellular conductivity	*σ_ex_*	0.14 S/m ^a^	29, 38
Intracellular conductivity	*σ_in_*	0.3 S/m ^b^	29, 39
Initial conductivity of membrane	*σ_m_*_0_	5e–7 S/m	40
Extracellular permittivity	*ɛ_o_*	7.1e–10 As/Vm	29
Intracellular permittivity	*ɛ_i_*	7.1e–10 As/Vm	29
Membrane permittivity	*ɛ_m_*	4.4e–11 As/Vm ^c^	29
Water relative dielectric constant	*ɛ_w_*	80 As/Vm	29
Lipid relative dielectric constant	*ɛ_l_*	2 As/Vm	29
Free diffusion coefficient	D_0_	5e–10 m^2^/s	29
Zero-field equilibrium constant	K_0_	2e–2	29
Mean average aqueous pore volume	Δ*V_p_*	9e–27 m^3^	29
Intrinsic barrier potential of P1 state	$\varphi_{\mathrm{im}1}^{0}$	0.13 V	31
Intrinsic barrier potential of P2 state	$\varphi_{\mathrm{im}2}^{0}$	0.084 V	31
A geometrical parameter	n	0.12	33
Decay rate coefficient for C1	k_−1_	10^5^ s^−1^	41–43
Decay rate coefficient for P1 pores	k_−2_	2000 s^−1^	41–43
Decay rate coefficient for P2 pores	k_−3_	2 s^−1^	41
Decay rate coefficients for endocytotic-like process	k_f_, k_s_	0.044, 0.003 s^−1^	29

a This is for SMEM. The range of extracellular medium is quite large.

b Reported between (0.2–0.55) S/m

c Reported between (4.4–5)*10^−11^ As/Vm
